# miR-203 and miR-221 regulate *SOCS1* and *SOCS3* in essential thrombocythemia

**DOI:** 10.1038/bcj.2016.10

**Published:** 2016-03-18

**Authors:** A Navarro, S Pairet, A Álvarez-Larrán, A Pons, G Ferrer, R Longarón, C Fernández-Rodríguez, L Camacho, M Monzó, C Besses, B Bellosillo

**Affiliations:** 1 Molecular Oncology and Embryology Laboratory, Human Anatomy Unit, School of Medicine, University of Barcelona, IDIBAPS, Barcelona, Spain; 2 Department of Pathology, Hospital del Mar, IDIBAPS, Barcelona, Spain; 3 Grup de Recerca Clínica Aplicada en Neoplàsies Hematològiques, IMIM (Hospital del Mar Medical Research Institute), IDIBAPS, Barcelona, Spain; 4 Department of Hematology, Hospital del Mar, IDIBAPS, Barcelona, Spain; 5 Institute of Hematology and Oncology, Hospital Clinic, IDIBAPS, Barcelona, Spain

## Abstract

The biological basis of essential thrombocythemia (ET) patients lacking known mutations is still unknown. MicroRNAs (miRNA) regulate hematopoietic differentiation and are deregulated in several hematopoietic malignancies. However, miRNA expression in ET patients has been poorly explored. We performed miRNA profiling in platelets from 19 ET patients and 10 healthy controls. Hierarchical cluster analysis showed two well-separated clusters between patients and controls, indicating that ET platelets had a characteristic 70-miRNA signature (*P*<0.0001), 68 of which were downregulated. According to the mutational status, three differentially expressed miRNAs, miR-15a (*P*=0.045), miR-150 (*P*=0.001) and miR-519a (*P*=0.036), were identified. A 40-miRNA signature was identified characterizing JAK2V617F-positive ET patients. Eight genes, whose interaction with the miRNAs could activate the JAK/STAT pathway were identified. An inverse correlation was observed between miRNAs expression and their target genes for *SOCS1* and miR-221, *SOCS3* and miR-221, *SOCS3* and miR-203, and PTPN11 and miR-23a. All three miRNAs were upregulated in JAK2V617F-negative ET patients. SOCS1 and SOCS3 were validated as targets of miR-221 and miR-203, respectively. In summary, our study shows that platelets from JAK2V617F-negative ET patients harbor a specific miRNA signature that can participate in the modulation of the JAK/STAT pathway through regulation of key genes as *SOCS1* and *SOCS3*.

## Introduction

Essential thrombocythemia (ET) is a chronic myeloproliferative neoplasm (MPN) characterized by sustained thrombocytosis, megakaryocytic proliferation and an increased tendency to thrombosis and bleeding.^1, 2^ The detection of the JAK2V617F mutation constitutes a key point in the diagnosis work-up of ET, being positive in 40–60% of the patients.^3^ This mutation, that has also been described in patients with polycythemia vera and primary myelofibrosis causes the constitutive activation of the JAK/STAT signaling pathway that is considered central to the pathogenesis and phenotype of MPN.^4^ Hyperactivation of the JAK/STAT pathway is not restricted to patients bearing the JAK2V617F mutation, and can also be observed in ET patients with mutations affecting the *MPL* gene that encodes the thrombopoietin receptor.^5, 6^ More recently, *CALR* mutations affecting the *CALR* gene have also been described in ET patients.^7, 8^ *CALR* codifies for the calreticulin protein, a chaperone located in the endoplasmic reticulum that has an important role in glycoprotein folding. Although not directly involved in the JAK/STAT pathway, cell lines transfected with mutant *CALR* show activated STAT5, however the mechanisms by which this signaling activation occurs remain unclear.^7, 8^ Nevertheless, a variable proportion of ET patients still lack a molecular marker.

MicroRNAs (miRNA) are short (18–24 nucleotides) non-coding RNAs that function primarily as gene repressors by binding to their target messenger RNAs (mRNAs).^9^ miRNAs regulate hematopoiesis in both hematopoietic stem cells and committed progenitor cells.^10^ Deregulated miRNAs have been reported in several hematological malignancies including MPNs.^11^ MiRNA studies in MPNs have been mostly performed in samples from polycythemia vera and primary myelofibrosis patients, but more limited information is available regarding ET patients.^12^ On the basis of our previous experience and from others, in ET, platelets show a higher clonal expansion than other cellular populations such as neutrophils and therefore molecular alterations are more easily detectable in this cellular population.^13, 14^ In the present work, we have studied the miRNA profile in platelets from JAK2V617F-positive and JAK2V617F-negative ET patients with the aim of characterizing the expression pattern of miRNAs involved in *JAK2*V617F-negative ET and identifying potential targets for these miRNAs that may explain the pathogenesis of the disease and be considered as potential biomarkers.

## Materials and methods

### Patients

Nineteen ET patients diagnosed according to World Health Organization criteria^15^ at the Hematology Department from Hospital del Mar were included in the study. Ten patients were *JAK2* V617F positive and nine *JAK2* V617F negative. From the nine *JAK2* V617F-negative patients, two harbored *CALR* mutations, two *MPL* mutations and five were triple-negative ET patients.

The samples of ET patients were collected before starting any treatment or received aspirin. Samples from 10 healthy controls were included as control group. The study was approved by the Clinical Research Ethics Committee Parc de Salut Mar and informed consent was obtained according to the Declaration of Helsinki.

### Molecular characterization

All patient samples were studied for *JAK2, MPL* and *CALR* mutations as previously described.^8, 16^ Briefly, JAK2V617F was assessed by allele-specific real-time PCR, *CALR* mutations were determined by amplification of exon 9 with fluorescently labeled primers followed by fragment analysis and *MPL* mutations were assessed by Sanger sequencing.

### Platelets isolation and RNA isolation

Platelets were isolated from peripheral blood as previously described.^13^ Briefly, 20 ml of venous blood was collected in ethylenediaminetetraacetic acid and immediately processed. Platelet-rich plasma was obtained by centrifugation of anticoagulated whole blood at 194 g for 10 min. Total RNA was extracted from isolated platelets using TRIzol reagent following the manufacturer's instructions (Life Technologies, Carlsbad, CA, USA).

### miRNA profiling

The expression of 384 mature miRNAs was quantified using TaqMan Human MicroRNA Arrays v2.0 (Life Technologies) as previously described.^17, 18^ Briefly, reverse transcription (RT) reaction was performed on Veriti 96-well thermal cycler for 2 min at 16 °C, 1 min at 42 °C and 1 s at 50 °C for 40 cycles, and 5 min at 85 ºC, and then held at 4 °C. The RT reaction contained: 0.80 μl of 10 × RT buffer (Life Technologies), 0.2 μl dNTPs (100 mM each), 1.5 μl MultiScribe Reverse Transcriptase (50 U/μl), 0.10 μl RNase Inhibitor (20 U/μl), 0.80 μl Megaplex RT primers (10 × ), 0.90 μl of MgCl_2_ (20 U/μl) and 500 ng of total RNA. Real-time PCR reaction was performed on an ABI 7900 HT Sequence Detection System (Life Technologies) and contained 450 μl of TaqMan Universal PCR Master Mix No Amperase (2 × ) (Life Technologies), 6 μl Megaplex RT product and 444 μl nuclease-free water.

### Normalization and filtering

The relative miRNA expression was calculated using the 2^−ΔΔCt^ method. Normalization was performed with RNU48 as after comparing the stability of RNU44, RNU48 and MammU6; RNU48 had the lowest variability of expression in the miRNA expression patient data set. All miRNAs expressed in <10% of samples were excluded from further analysis, leaving a working set of 273 miRNAs.

### miRNA target selection and validation

To identify molecular pathways potentially altered by the expression of multiple miRNAs we used Diana-mirPath,^19^ which performs an enrichment analysis of multiple miRNA target genes, comparing each set of miRNA targets to all known kyoto encyclopedia of genes and genomes pathways. After that, mRNA expression of putative selected targets was analyzed using TaqMan gene expression assays (Life Technologies). The genes, whose expression was negatively correlated with miRNAs, were selected for further target validation by Renilla/luciferase assay and western Blot.

### Renilla/luciferase assay

Cloning of the target sequence was performed as previously described.^17, 20^ Briefly, two synthetic oligonucleotides containing the 3′ untranslated region (3′UTR) target sequence for each studied gene (Supplementary Table 1) were cloned in the 3′UTR region of *Renilla luciferase* gene in the psiCHECK-2 vector (Promega, Madison, WI, USA) using NotI and XhoI restriction sites.

For Renilla luciferase assay 100 nM pre-miRNAs where transfected in K562 cell line together with 0.2 μg of modified psicheck2 vector and Renilla luciferase levels were measured at 24 h after transfection using a Promega Dual luciferase reporter assay system (Promega) in an Orion II microplate luminometer (Berthold Detection Systems GmbH, Pforzheim, Germany). The transfection efficiency was normalized with the Firefly luciferase gene.

### Western blot

Transfected cells were lysed in 1% RIPA buffer, 62.5 mM Tris HCl 1 M pH=6.8, 5% β-mercaptoethanol, 2% sodium dodecyl sulfate, 40% glycerol, 0.005% bromophenol blue and equal amounts of protein were separated by electrophoresis on 12% polyacrilamide gel and transferred to Immobilion-P (Millipore, Bedford, MA, USA) membranes. The membranes were incubated with polyclonal antibody against SOCS1, SOCS3 (Abcam, Cambridge, UK) and α-tubulin (Sigma, St Louis, MO, USA). Antibody binding was detected using a secondary antibody (mouse anti-rabbit and mouse anti-mouse immunoglobulin (Dako, Glostrup, Denmark) conjugated to horseradish peroxidase and an enhanced chemiluminiscence detection kit (Amersham, Buckinghamshire, UK).

### Statistical analysis

Data from miRNA expression were analyzed using TIGR Multiexperiment viewer version 4.0 software (Dana-Farber Cancer Institute, Boston, MA, USA), BRB Array Tools (Biometric Research Branch, National Cancer Institute, National Institutes of Health; http://linus.nci.nih.gov/BRB-ArrayTools.html), GraphPad Prism 5 and SPSS 15 (SPSS Inc., Chicago, IL, USA). Class comparison and Student's *t*-test were used to analyze differences between groups. Characteristics between groups were compared using the *χ*^2^-test and Fisher's exact test, when applicable, for categorical variables, and *t*-test for continuous variables, respectively. A two-sided *P*-value<0.05 was considered statistically significant.

## Results

### Profiling miRNA expression in ET patients

We performed miRNA profiling of platelets from 29 cases included in the study by real-time PCR using arrays that allow simultaneous analysis of 384 miRNAs. After filtering and normalization, 273 miRNAs were left for further analysis.

**Figure 1:**
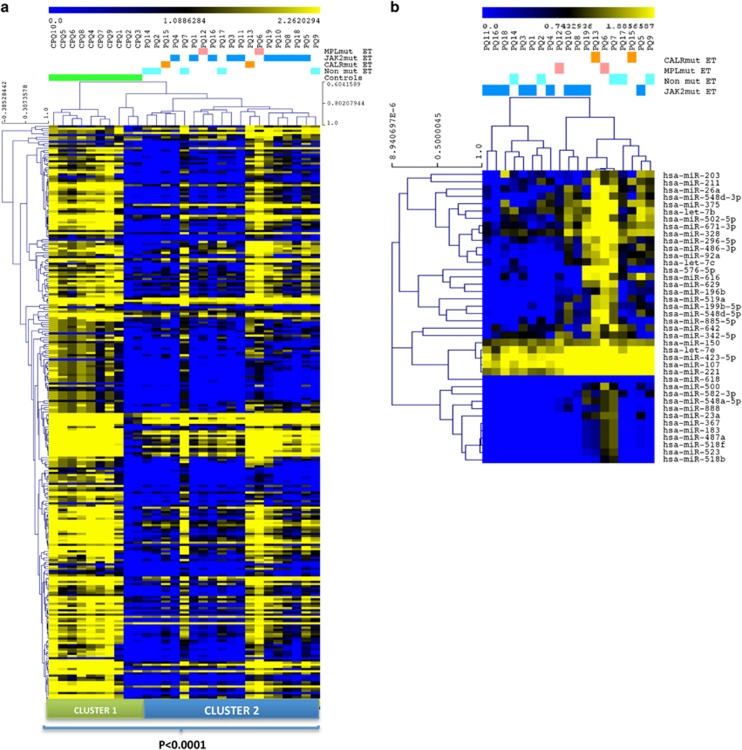
miRNA expression pattern in platelets from ET patients and healthy controls. (**a**) Unsupervised hierarchical cluster analysis including all samples. (**b**) Hierarchical cluster analysis of the 40 miRNAs identified by significance analysis of microarrays analysis that were differentially expressed between the *JAK2*V617F (JAK2-mut) vs *JAK2*-wild-type ET patients.

The unsupervised hierarchical cluster analysis of platelet miRNA profile showed two well-separated clusters between ET patients and controls, indicating that ET platelets had a characteristic miRNA signature (*P*<0.0001; Figure 1a). The supervised analysis showed that ET patients harbored a distinctive signature of 70 miRNAs, 68 of which were downregulated (Supplementary Table 2). Only miR-9 (*P*=0.005) and miR-431 (*P*=0.007) were significantly upregulated in ET patients.

We then identified miRNAs differentially expressed in ET patients according to the mutational status. Using one-way analysis of variance based on multiple permutations, we identified three miRNAs whose expression was significantly different between *JAK2*-mutant, *CALR*-mutant, *MPL*-mutant and triple-negative ET patients: miR-15a (*P*=0.045), miR-150 (*P*=0.001) and miR-519a (*P*=0.036) (Supplementary Figure 1).

Finally, we analyzed miRNA expression according to clinical characteristics of the patients (age, leukocyte number, platelet number and hemoglobin levels) using Quantitative trait analysis by mean of Spearman correlation (*P*<0.01). Six miRNAs showed a negative correlation with hemoglobin levels: miR-874 (*r*=−6.62, *P*=0.002), miR-500 (*r*=−0.646, *P*=0.003), miR-196b (*r*=−0.644, *P*=0.003), miR-200a (*r*=−0.618, *P*=0.05), miR-365 (*r*=−0.596, *P*=0.008) and miR-429 (*r*=-0.596, *P*=0.008). Eighty-eight miRNAs were correlated with platelet number (Supplementary Table 3) including miR-499-5p (*r*=0.76, *P*=0.0002), miR-424 (*r*=0.74, *P*=0.0003), miR-509-5p (*r*=0.71, *P*=0.00008) and miR-886-5p (*r*=0.71, *P*=0.0008) as the most significantly correlated. No correlation with age and leukocyte number was observed.

### Identification of a miRNA signature associated with the JAK/STAT pathway

**Figure 2:**
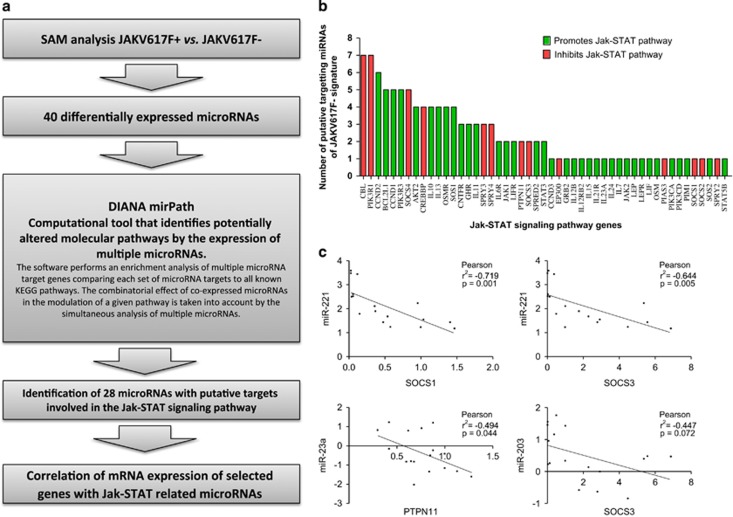
Identification of miRNAs targeting the JAK/STAT pathway. (**a**) Pipeline used to identify miRNAs targeting the JAK/STAT pathway. (**b**) Bar graph showing the JAK/STAT-related genes identified (*x* axis) and the number of miRNAs potentially targeting each of these genes (*y* axis). (**c**) Correlation graph of the four miRNA gene pairs identified: *SOCS1* and miR-221; *SOCS3* and miR-221; *SOCS3* and miR-203; and *PTPN11* and miR-23a.

To identify a miRNA signature regulating the JAK2 pathway, we compared the 10 JAK2V617F-mutated vs the nine *JAK2* wild-type patients. Supervised significance analysis of microarrays analysis identified 40 miRNAs that were differentially expressed between the two groups (Figures 1b and 2a; Table 1). We then performed an *in silico* analysis to test if these 40 miRNAs regulated the JAK/STAT pathway. Using Diana-mirPath,^19^ we performed an enrichment analysis (Figure 2a) to identify the set of miRNAs acting together in the regulation of the JAK2 pathway. Interestingly, we identified 28 miRNAs (bold highlighted miRNAs in Table 1) with putative targets involved in the JAK/STAT signaling pathway. Figure 2b shows the JAK/STAT-related genes identified and the number of miRNAs potentially targeting each of these genes.

### Validation of the *in silico* analysis: miR-221 and miR-203 target SOCS1 and SOCS3

**Figure 3:**
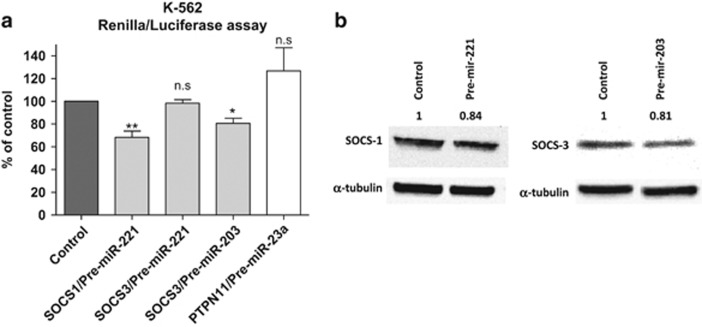
Target validation study by (**a**) Renilla luciferase assay and (**b**) western blot in K562 transfected cells.

To validate the *in silico* analysis, we selected eight genes whose interaction with the predicted miRNAs could activate the JAK/STAT pathway in the JAK2 wild-type patients: *CBL, CCND1, SOCS1, SOCS2, SOCS3, SOCS4, PTPN11* and *BCL2L1*. Next, we analyzed the expression of the selected genes by quantitative real-time PCR to identify any correlation between gene and miRNA expression. We found a significant inverse correlation in four miRNA gene pairs (Figure 2c): *SOCS1* and miR-221 (*r*^2^=−0.719, *P*=0.001); SOCS3 and miR-221 (*r*^2^=−0.644, *P*=0.005); *SOCS3* and miR-203 (*r*^2^=−0.447, *P*=0.072) and *PTPN11* and miR-23a (*r*^2^=−0.494, *P*=0.044). All three miRNAs were upregulated in *JAK2* wild-type patients in comparison with *JAK2*V617F-mutant patients. To validate these target genes, we cloned them and performed Renilla luciferase assays. These experiments confirmed *SOCS1* as a target of miR-221 (28.9% Renilla luciferase protein reduction, *P*=0.002) and *SOCS3* as a target of miR-203 (19.6% Renilla luciferase protein reduction, *P*=0.04; Figure 3a). No significant modifications were observed for *PTPN11*. Further validation of *SOCS1* and *SOCS3* was performed by western blot that showed a significant reduction of the protein levels of SOCS1 (16%) and SOCS3 (19%), after increasing the levels of miR-221 and miR-203, respectively (Figure 3b).

## Discussion

There is growing evidence that miRNAs are involved in the regulation of hematopoiesis.^12^ However, our understanding of the role of miRNAs in MPN pathogenesis is still limited. We have aimed at analyzing the miRNA expression profiling in platelets from ET patients. Although platelets are anucleated, they retain the capacity for protein synthesis, as well as a competent miRNA pathway capable of converting precursor miRNAs to mature forms that can modulate, among others, the expression of the thrombopoietin receptor.^21^

Our results have shown that platelets from ET patients harbor a distinctive signature of miRNAs when compared with healthy controls. This observation is in line with the work reported by Xu *et al.*^22^ that analyzed miRNA expression patterns in cases with thrombocytosis and compared them with normal controls. In this line, several miRNAs reported by Xu *et al.* in ET vs healthy controls agree with our results, including miR-9, miR-181c, miR-150 and miR-182. Moreover, in Xu's work a specific signature was associated with increased megakariopoiesis (in both reactive thrombocytosis and ET), and among the differentially expressed miRNAs an increased expression of miR-490-5p was observed. In addition, this was also associated with a disregulation of one of the putative targets of miR-490-5p, the *DAAM1* (disheveled associated activator of morphogenesis 1) gene. Unfortunately, we cannot corroborate this result as this miRNA was not included in the array used in our study to profile miRNAs. Although we could not analyze the expression of miR-490-5p, these authors also described disregulation of miR-150. miR-150 is expressed in megakaryocyte–erythoid (MEP) progenitor cells and its overexpression commits MEPs towards megakaryocyte in normal hematopoiesis.^23^ In agreement with Xu, we found this miRNA as one of the most heavily downregulated in ET patients compared to normal controls. Interestingly, we have observed that miR-150 expression varies according the mutational status in ET patients, where MPLmut patients had the higher levels and JAK2mut patients had the lower levels. Moreover, miR-150 is one of the miRNAs composing our signature of JAK2mut vs JAK2 non-mutated patients and have interesting putative targets of the JAK2 pathway such as *CBL*, *EP300* or *PIK3R1* (Table 1) and validated targets such as STAT1.^24^

The pathogenetic hallmark of MPNs is the hyperactivation of the JAK/STAT signaling pathway.^25, 26^ This deregulation is usually associated with the *JAK2*V617F mutation, but also with mutations in the *CALR* and *MPL* genes that are also involved in the development of MPNs, and specifically in ET.^7, 27, 28^ In the present work, we focused in the identification of miRNAs of the JAK/STAT pathway associated with the *JAK2*V617F mutation. We have analyzed whether miRNAs differentially expressed between *JAK2*V617F-positive and -negative patients, could account for an activation of the JAK/STAT pathway in patients lacking the V617F mutation. Among the miRNAs identified with putative targets involved in the JAK/STAT signaling pathway (*n*=28, Table 1), a significant inverse correlation between miR-203 and miR-221 expression was found with *SOCS1* and *SOCS3* genes, which are negative regulators of the JAK/STAT pathway and we validated the targeting by Renilla/luciferasa assay and western blot. Silencing of *SOCS1* and *SOCS3* have been previously related to MPN but the mechanism of silencing it is not completely clear. Hypermethylation of CpG islands in *SOCS1* and *SOCS3* associated with a decrease in expression was found in JAK2V617F polycythemia vera and ET as well as in *JAK2*V617F and *MPL*W515-mutation negative ET.^29^ However, other authors could not confirm the hypermethylation of these genes although they observed differences in the gene expression pattern among MPN in a significant proportion of patients with idiopathic myelofibrosis but not in patients with polycythemia vera or ET.^30, 31^ Recently, Jost *et al.*^32^ reported methylation of *SOCS1* in 15% of MPD patients. *SOCS1* expression was increased, to varying degrees, in most types of MPD.^29, 32, 33^ In this line, miRNAs alone or in combination with methylation processes, could be explaining the downregulation of *SOCS1* and *SOCS3* in the ET JAK2V617F-negative patients and participating by this way in the activation of the JAK2 pathway. In the same line of our results, miR-203 and miR-221 have been reported regulating *SOCS3* and *SOCS1* in other pathologies. In breast cancer miR-203 participates in the chemoresistance to cisplatin through the direct regulation of *SOCS3.*^34^ The induction of miR-203 expression by *Porphyromonas gingivalis* in gingival epithelial cells inhibits *SOCS3* and activates *STAT3.*^35^ *Helicobacter pylori* causes hepatic insulin resistance through regulation of miR-203 levels that modulates *SOCS3* levels.^36^ Finally, in hepatocelular carcinoma it has been reported that miR-221 regulates *SOCS1* and *SOCS3* and this accentuates IFN's anti-HCV effect,^37^ although in our study we only found association of miR-221 and *SOCS1* but not with *SOCS3*. Interestingly, miR-203 has also been reported to be silenced by methylation in *BCR-ABL1* positive cells from CML patients.^38^ The study of the methylation grade of these miRNAs in MPN warrants further investigation.

In summary, we have reported in the present work a 40-miRNA signature that characterizes JAK2V617F-negative platelets from ET patients. The analysis of the putative targets of the miRNAs of this signature allowed us to identify two miRNAs, miR-221 and miR-203, targeting *SOCS1* and *SOCS3* that are negative regulators of the JAK/STAT pathway. The upregulation of these miRNAs could be one of the factors involved in the activation of this signaling pathway in JAK2V617F-negative ET patients. The identification of miRNAs involved in the regulation of the JAK/STAT pathway in patients harboring other mutations such as *CALR* and *MPL* would be valuable to increase the actual knowledge of the mechanisms involved in the pathogenesis of ET, but in the present work the low number of patients who harbored that mutations prevented the completion of the analysis. Further investigation is warranted to shed light on the role of the miRNAs in ET.

## Figures and Tables

**Table 1:** List of miRNAs differentially expressed between JAK2V617F-positive and -negative ET patients

*microRNA*	*Fold change*	*Predicted targets of JAK/STAT signaling pathway (DIANA LAB)*
**hsa-miR-576-5p**	**1.9475387**	**IL10, LEPR, SOCS2, SOCS4, SPRY4**
hsa-miR-885-5p	1.7738975	—
**hsa-miR-519a**	**1.7664691**	**JAK1, OSM, PIK3R1, SOS1, SPRY4, STAT3**
**hsa-miR-618**	**1.7017285**	**IL13**
hsa-miR-518b-	1.6955159	—
hsa-miR-548d-5p	1.6558107	—
**hsa-miR-221**	**1.6522014**	**CBL, PIK3R1, SOCS1, SOCS3, SPRED2**
**hsa-let-7c**	**1.6507319**	**AKT2, BCL2L1,CBL, CCND1, CCND2, GHR, IL10, IL13, OSMR, SOCS4**
hsa-miR-328	1.6371825	—
**hsa-miR-629**	**1.6326199**	**SPRY3**
**hsa-miR-211**	**1.6268274**	**CCND2, IL12RB2, IL23A, JAK2, PTPN11, SOS1**
**hsa-miR-196b**	**1.6259091**	**OSMR, SOCS4**
hsa-miR-671-3p	1.6118121	—
**hsa-miR-26a**	**1.6108524**	**CCND2, CREBBP, LIF, LIFR, PIK3R3, PIM1**
**hsa-miR-23a**	**1.598495**	**CCND1, CREBBP, IL11, IL12B, IL21R, IL6R, JAK1, PIK3R3, PTPN11, SOS1, SPRY2, STAT5B**
**hsa-miR-92a**	**1.5960894**	**PIK3R3**
**hsa-miR-548d-3p**	**1.5926827**	**CCND1, CCND2, CREBBP, GRB2, IL11, IL6R, LIFR, PIK3R1, PIK3R3, SPRY4**
**hsa-miR-642**	**1.5810314**	**CCND3, PIK3R1,**
**hsa-miR-296-5p**	**1.5807419**	**CNTFR, LEP**
**hsa-miR-616**	**1.5796043**	**PIK3R1**
hsa-miR-375	1.579199	—
hsa-miR-500	1.573965	—
hsa-miR-523	1.5546302	—
**hsa-miR-107**	**1.5477945**	**PIK3R1, SOS1, SPRY3**
**hsa-let-7b**	**1.5216084**	**AKT2, BCL2L1, CBL, CCND1, CCND2, GHR, IL10, IL13, OSMR, SOCS4**
hsa-miR-888	1.5203407	—
44hsa-miR-518f	1.5167831	—
**hsa-let-7e**	**1.5159571**	**AKT2, BCL2L1, CBL, CCND1, CCND2, GHR, IL10, IL13, OSMR, SOCS4**
**hsa-miR-487a**	**1.5127604**	**SPRED2**
**hsa-miR-183**	**1.5127604**	**SPRY3**
**hsa-miR-367**	**1.5127604**	**PIK3R3**
**hsa-miR-582-3p**	**1.5057397**	**CREBBP**
hsa-miR-423-5p	1.4989724	—
**hsa-miR-199b-5p**	**1.4976261**	**CBL, PIK3CD, SOS2**
**hsa-miR-486-3p**	**1.45879**	**CNTFR**
hsa-miR-502-5p	1.4474875	—
**hsa-miR-548a-5p**	**1.4405084**	**IL11, IL7, PIAS3, STAT3**
**hsa-miR-342-5p**	**1.4166621**	**BCL2L1**
**hsa-miR-203**	**1.3055979**	**AKT2, CBL, CNTFR, IL15, IL24, PIK3CA, SOCS3**
**hsa-miR-150**	**1.1759403**	**CBL, EP300, PIK3R1**

MiRNAs are ordered by fold change. The miRNAs with putative targets from the JAK/STAT pathway are indicated in bold and the putative target genes from the JAK/STAT pathway are included.
