# Supplementary material for: miR-203 and miR-221 regulate SOCS1 and SOCS3 in essential thrombocythemia
Source: Blood Cancer J. 2016 Mar 18;6(3):e406–. doi: 10.1038/bcj.2016.10 (PMC4817095; doi:10.1038/bcj.2016.10)
Supplement: Supplementary Table 1 [file bcj201610x1.docx]

**Supplementary Table 1. Oligonucleotides used in the preparation of the modified psiCheck2 vectors used in the Renilla/Luciferase study.**

| **Gene / miRNA** | **Oligonucletotides** |
| --- | --- |
| **SOCS1 / miR-221** | 5’- TCGAGTCCCTCTGGGTCCCCCTGGTTGTTGTAGCAGCTTAACTGTATCTGGAAGATCTGC-3’  5’- GGCCGCAGATCTTCCAGATACAGTTAAGCTGCTACAACAACCAGGGGGACCCAGAGGGAC -3’ |
| **SOCS3 / miR-221** | 5’- TCGAGTAGCACTGATCAGTGACAATTTACAGGAATGTAGCAGCGATGGAATTACCTGGAACAAGATCTGC-3’  5’- GGCCGCAGATCTTGTTCCAGGTAATTCCATCGCTGCTACATTCCTGTAAATTGTCACTGATCAGTGCTAC-3’ |
| **SOCS3 / miR-203** | 5’- TCGAGACTTTGCACATATTTATATTTATATTCAGAAAAGAAACATTTCAGTAATTTATAATAGATCTGC-3’  5’- GGCCGCAGATCTATTATAAATTACTGAAATGTTTCTTTTCTGAATATAAATATAAATATGTGCAAAGTC -3’ |
| **PTPN11 / miR-23a** | F5’- TCGAGGGAATACGTTTTAGGATGTCATCATTTTGATGTGAATCATGTAAATGTTGATAAAGATCTGC-3’  5’- GGCCGCAGATCTTTATCAACATTTACATGATTCACATCAAAATGATGACATCCTAAAACGTATTCCC -3’ |
