# Supplementary material for: miR-203 and miR-221 regulate SOCS1 and SOCS3 in essential thrombocythemia
Source: Blood Cancer J. 2016 Mar 18;6(3):e406–. doi: 10.1038/bcj.2016.10 (PMC4817095; doi:10.1038/bcj.2016.10)
Supplement: Supplementary Table 2 [file bcj201610x2.docx]

**Supplementary Table 2. List of miRNAs differentially expressed between ET patients and healthy control samples.**

| **miRNA** | **Control mean** | **Control STD** | **ET mean** | **ET STD** | **Raw p- value** | **Adj p-value** | **FDR** |
| --- | --- | --- | --- | --- | --- | --- | --- |
| hsa-miR-182 | 2.1255462 | 1.3974197 | 0.13248615 | 0.7123228 | <0.001 | <0.001 | <0.01 |
| hsa-miR-133a | 3.8898418 | 0.9465643 | 2.2527664 | 0.8021894 | <0.001 | <0.001 | <0.01 |
| hsa-miR-150 | 1.7109957 | 0.17705613 | 1.3003412 | 0.221236 | <0.001 | <0.001 | <0.01 |
| hsa-miR-203 | 1.9380617 | 0.7773723 | 0.38688117 | 0.8215966 | <0.001 | <0.001 | <0.01 |
| hsa-miR-95 | 1.3602605 | 0.5657763 | 0.5254884 | 0.5190878 | <0.001 | <0.001 | <0.01 |
| hsa-miR-190 | 1.2800429 | 1.5038122 | -0.43432888 | 0.981714 | 0.001 | 0.001 | 0.04466667 |
| hsa-miR-196b | 1.332617 | 0.86799407 | 0.078026354 | 0.6845279 | 0.002 | 0.002 | 0.067 |
| hsa-miR-518e | 1.0533593 | 1.1699873 | -0.35452533 | 0.6571622 | 0.002 | 0.002 | 0.067 |
| hsa-miR-590-5p | 1.3014818 | 0.7823931 | 0.072188854 | 0.9809174 | 0.002 | 0.002 | 0.067 |
| hsa-miR-200a | 1.5299971 | 0.93042845 | 0.14744216 | 0.80687 | 0.002 | 0.002 | 0.067 |
| hsa-miR-888 | 1.141517 | 1.0977659 | -0.2595316 | 0.6931028 | 0.002 | 0.002 | 0.067 |
| hsa-miR-1 | 3.2420406 | 1.1602825 | 1.7150104 | 0.8648455 | 0.002 | 0.002 | 0.067 |
| hsa-miR-504 | 1.4620711 | 1.5201262 | -0.36462155 | 0.7361432 | 0.002 | 0.002 | 0.067 |
| hsa-miR-484 | 2.5122077 | 1.1474706 | 1.0008137 | 0.740365 | 0.002 | 0.002 | 0.067 |
| hsa-miR-582-3p | 1.2018721 | 1.1940761 | -0.18388522 | 0.7921221 | 0.003 | 0.003 | 0.05025 |
| hsa-miR-339-3p | 3.118566 | 0.87622625 | 1.9633068 | 0.7296746 | 0.003 | 0.003 | 0.05025 |
| hsa-miR-192 | 0.87625515 | 0.87507546 | -0.2970102 | 0.7341302 | 0.003 | 0.003 | 0.05025 |
| hsa-miR-193a-3p | 1.0413558 | 0.9615509 | -0.20800678 | 0.6298308 | 0.003 | 0.003 | 0.05025 |
| hsa-miR-199a-3p | 4.13896 | 1.40709 | 2.6999254 | 0.7814114 | 0.003 | 0.003 | 0.05025 |
| hsa-miR-125a-5p | 2.2699099 | 0.9837941 | 1.1006985 | 0.5866439 | 0.003 | 0.003 | 0.05025 |
| hsa-miR-126 | 4.111623 | 1.2245102 | 2.6858897 | 0.8007501 | 0.003 | 0.003 | 0.05025 |
| hsa-miR-345 | 1.6884186 | 1.104323 | 0.32537332 | 0.7834876 | 0.004 | 0.004 | 0.04466667 |
| hsa-miR-218 | 1.1817535 | 1.1607028 | -0.24871728 | 0.73489 | 0.004 | 0.004 | 0.04466667 |
| hsa-miR-545 | 1.2181392 | 1.2538949 | -0.31638965 | 0.6882483 | 0.004 | 0.004 | 0.04466667 |
| hsa-miR-143 | 1.7965664 | 1.5308632 | 0.12532096 | 0.9233382 | 0.004 | 0.004 | 0.04466667 |
| hsa-miR-618 | -0.03297744 | 1.3845465 | -1.661587 | 0.6729423 | 0.004 | 0.004 | 0.04466667 |
| hsa-miR-138 | 2.1749425 | 0.93863875 | 0.829628 | 1.1455013 | 0.004 | 0.004 | 0.04466667 |
| hsa-miR-140-3p | 1.1015279 | 0.9064002 | 0.008880432 | 0.6959245 | 0.004 | 0.004 | 0.04466667 |
| hsa-miR-191 | 1.986715 | 0.982247 | 0.8366959 | 0.744003 | 0.004 | 0.004 | 0.04466667 |
| hsa-miR-326 | 2.880196 | 1.439081 | 1.3758326 | 0.7923673 | 0.005 | 0.005 | 0.04060606 |
| hsa-miR-454 | 1.6297512 | 0.8888196 | 0.59811515 | 0.7299444 | 0.005 | 0.005 | 0.04060606 |
| hsa-miR-330-5p | 1.1539757 | 1.2488912 | -0.35573488 | 0.7062514 | 0.005 | 0.005 | 0.04060606 |
| hsa-miR-9 | 0.24320328 | 0.56121045 | 1.2009908 | 0.9873703 | 0.005 | 0.005 | 0.04060606 |
| hsa-miR-367 | 0.93987024 | 1.1160911 | -0.38526365 | 0.6750138 | 0.005 | 0.005 | 0.04060606 |
| hsa-miR-548b-5p | 0.011728731 | 1.1160911 | -1.1931996 | 0.7650411 | 0.005 | 0.005 | 0.04060606 |
| hsa-miR-183 | 0.93987024 | 1.1160911 | -0.38526365 | 0.6750138 | 0.005 | 0.005 | 0.04060606 |
| hsa-miR-216b | 0.93987024 | 1.1160911 | -0.31634036 | 0.6510283 | 0.005 | 0.005 | 0.04060606 |
| hsa-miR-487a | 0.93987024 | 1.1160911 | -0.38526365 | 0.6750138 | 0.005 | 0.005 | 0.04060606 |
| hsa-miR-582-5p | -0.65627533 | 1.1339494 | -1.8539913 | 0.754287 | 0.006 | 0.006 | 0.03921951 |
| hsa-miR-876-3p | 0.93987024 | 1.1160911 | -0.34401888 | 0.7262308 | 0.006 | 0.006 | 0.03921951 |
| hsa-miR-324-5p | 2.2305436 | 1.2576888 | 0.78845334 | 1.0584772 | 0.006 | 0.006 | 0.03921951 |
| hsa-miR-873 | 0.93987024 | 1.1160911 | -0.2850954 | 0.6561546 | 0.007 | 0.007 | 0.03752 |
| hsa-miR-324-3p | 1.9604737 | 1.2836345 | 0.6311342 | 0.7330733 | 0.007 | 0.007 | 0.03752 |
| hsa-miR-518b | 0.6672319 | 1.1160911 | -0.50052524 | 0.6562532 | 0.007 | 0.007 | 0.03752 |
| hsa-miR-186 | 1.8624694 | 1.0212201 | 0.73263395 | 0.8603628 | 0.007 | 0.007 | 0.03752 |
| hsa-miR-523 | 1.0470995 | 1.3536407 | -0.52748007 | 0.6932965 | 0.007 | 0.007 | 0.03752 |
| hsa-miR-518f | 1.0954775 | 1.3047622 | -0.38841116 | 0.6788775 | 0.007 | 0.007 | 0.03752 |
| hsa-miR-340 | 2.1111226 | 1.270081 | 0.79920435 | 0.895941 | 0.007 | 0.007 | 0.03752 |
| hsa-miR-34c-5p | 0.4023734 | 1.1162533 | -0.85912895 | 0.7012049 | 0.007 | 0.007 | 0.03752 |
| hsa-miR-106b | 1.5822604 | 1.2736436 | 0.27171555 | 0.8353332 | 0.007 | 0.007 | 0.03752 |
| hsa-miR-431 | 2.0105772 | 1.2182963 | 3.3962483 | 0.7995683 | 0.007 | 0.007 | 0.03752 |
| hsa-miR-136 | 1.046114 | 1.0174448 | -0.13259299 | 0.7827249 | 0.007 | 0.007 | 0.03752 |
| hsa-miR-181c | 1.8944035 | 1.4118106 | 0.43029734 | 0.8840995 | 0.008 | 0.008 | 0.03970371 |
| hsa-miR-133b | 2.3874984 | 1.4367388 | 0.9637445 | 0.7939585 | 0.008 | 0.008 | 0.03970371 |
| hsa-miR-202 | 0.98464376 | 1.6031927 | -0.6485894 | 0.6797355 | 0.008 | 0.008 | 0.03970371 |
| hsa-miR-16 | 1.0952234 | 1.0089239 | 0.043841153 | 0.7507011 | 0.008 | 0.008 | 0.03970371 |
| hsa-miR-570 | 0.6307536 | 0.9487747 | -0.3958423 | 0.9862407 | 0.009 | 0.009 | 0.04158621 |
| hsa-miR-651 | 1.0268558 | 1.1421201 | -0.23008446 | 0.8127469 | 0.009 | 0.009 | 0.04158621 |
| hsa-miR-146b-5p | 2.0593622 | 0.7828295 | 1.18922 | 0.7578075 | 0.009 | 0.009 | 0.04158621 |
| hsa-miR-223 | 0.9708818 | 1.1500517 | -0.1495512 | 0.7153871 | 0.009 | 0.009 | 0.04158621 |
| hsa-miR-518d-3p | 1.037718 | 1.102769 | -0.24191144 | 0.9228383 | 0.009 | 0.009 | 0.04158621 |
| hsa-miR-451 | -0.41665664 | 1.3897948 | -1.7378975 | 0.5161073 | 0.009 | 0.009 | 0.04158621 |
| hsa-miR-519e | 1.1993771 | 1.1160911 | -0.00518008 | 0.8371506 | 0.009 | 0.009 | 0.04158621 |
| hsa-miR-335 | 3.4145107 | 1.7218121 | 1.6907479 | 0.8321826 | 0.01 | 0.01 | 0.04 |
| hsa-miR-331-5p | 2.1481051 | 1.620065 | 0.63396776 | 0.9190694 | 0.01 | 0.01 | 0.04 |
| hsa-miR-124 | -0.39049095 | 1.1160911 | -1.5589025 | 0.6629255 | 0.01 | 0.01 | 0.04 |
| hsa-miR-212 | 1.2452633 | 0.84666467 | 0.30343208 | 0.8181834 | 0.01 | 0.01 | 0.04 |
| hsa-miR-548a-5p | 0.938303 | 1.1161367 | -0.21069919 | 0.7565497 | 0.01 | 0.01 | 0.04 |
| hsa-miR-301b | 3.060453 | 1.2325649 | 1.8661501 | 0.8231241 | 0.01 | 0.01 | 0.04 |
| hsa-miR-26b | 1.8392102 | 1.518637 | 0.37773234 | 0.8647801 | 0.01 | 0.01 | 0.04 |
