# Supplementary material for: miR-203 and miR-221 regulate SOCS1 and SOCS3 in essential thrombocythemia
Source: Blood Cancer J. 2016 Mar 18;6(3):e406–. doi: 10.1038/bcj.2016.10 (PMC4817095; doi:10.1038/bcj.2016.10)
Supplement: Supplementary Table 3 [file bcj201610x3.docx]

**Supplementary Table 3. miRNAs correlated with platelet count.**

| **N** | **Correlation coefficient** | **Parametric p-value** | **microRNA** |
| --- | --- | --- | --- |
| 1 | 0.76 | 0.0002454 | hsa-miR-499-5p |
| 2 | 0.747 | 0.0003556 | hsa-miR-424 |
| 3 | 0.714 | 0.0008528 | hsa-miR-509-5p |
| 4 | 0.712 | 0.0008893 | hsa-miR-886-5p |
| 5 | 0.69 | 0.0014891 | hsa-miR-490-3p |
| 6 | 0.684 | 0.0016656 | hsa-miR-9 |
| 7 | 0.682 | 0.0017281 | hsa-miR-548d-3p |
| 8 | 0.682 | 0.0017281 | hsa-miR-636 |
| 9 | 0.681 | 0.0017924 | hsa-miR-625 |
| 10 | 0.677 | 0.001927 | hsa-miR-362-5p |
| 11 | 0.67 | 0.0022208 | hsa-miR-574-3p |
| 12 | 0.665 | 0.0024643 | hsa-miR-363 |
| 13 | 0.665 | 0.0024643 | hsa-miR-660 |
| 14 | 0.661 | 0.0026383 | hsa-miR-132 |
| 15 | 0.66 | 0.0027291 | hsa-miR-449b |
| 16 | 0.66 | 0.0027291 | hsa-miR-589 |
| 17 | 0.654 | 0.0030166 | hsa-miR-96 |
| 18 | 0.651 | 0.0032217 | hsa-miR-197 |
| 19 | 0.646 | 0.0035506 | hsa-miR-138 |
| 20 | 0.646 | 0.0035506 | hsa-miR-671-3p |
| 21 | 0.644 | 0.0036661 | hsa-miR-128 |
| 22 | 0.644 | 0.0036661 | hsa-miR-330-3p |
| 23 | 0.644 | 0.0036661 | hsa-miR-409-5p |
| 24 | 0.64 | 0.0039064 | hsa-miR-34a |
| 25 | 0.639 | 0.0040313 | hsa-miR-362-3p |
| 26 | 0.639 | 0.0040313 | hsa-miR-425 |
| 27 | 0.639 | 0.0040313 | hsa-miR-486-5p |
| 28 | 0.633 | 0.0044257 | hsa-miR-215 |
| 29 | 0.632 | 0.004564 | hsa-miR-191 |
| 30 | 0.632 | 0.004564 | hsa-miR-532-3p |
| 31 | 0.628 | 0.0048511 | hsa-miR-148a |
| 32 | 0.628 | 0.0048511 | hsa-miR-339-5p |
| 33 | 0.628 | 0.0048511 | hsa-miR-548b-5p |
| 34 | 0.626 | 0.0050001 | hsa-miR-193a-5p |
| 35 | 0.625 | 0.0051528 | hsa-miR-532-5p |
| 36 | 0.623 | 0.0053093 | hsa-miR-484 |
| 37 | 0.621 | 0.0054697 | hsa-miR-106a |
| 38 | 0.621 | 0.0054697 | hsa-miR-579 |
| 39 | 0.621 | 0.0054697 | hsa-miR-93 |
| 40 | 0.619 | 0.005634 | hsa-miR-21 |
| 41 | 0.619 | 0.005634 | hsa-miR-22 |
| 42 | 0.619 | 0.005634 | hsa-miR-374a |
| 43 | 0.619 | 0.005634 | hsa-miR-486-3p |
| 44 | 0.618 | 0.0058023 | hsa-miR-139-5p |
| 45 | 0.618 | 0.0058023 | hsa-miR-449a |
| 46 | 0.616 | 0.0059748 | hsa-miR-210 |
| 47 | 0.616 | 0.0059748 | hsa-miR-223 |
| 48 | 0.612 | 0.0063322 | hsa-miR-17 |
| 49 | 0.61 | 0.0065173 | hsa-miR-491-5p |
| 50 | 0.609 | 0.0067068 | hsa-miR-27b |
| 51 | 0.609 | 0.0067068 | hsa-miR-30b |
| 52 | 0.605 | 0.0070994 | hsa-let-7a |
| 53 | 0.605 | 0.0070994 | hsa-miR-221 |
| 54 | 0.604 | 0.0073026 | hsa-miR-130a |
| 55 | 0.604 | 0.0073026 | hsa-miR-503 |
| 56 | 0.604 | 0.0073026 | hsa-miR-651 |
| 57 | 0.602 | 0.0075106 | hsa-miR-518d-3p |
| 58 | 0.6 | 0.0077233 | hsa-miR-139-3p |
| 59 | 0.6 | 0.0077233 | hsa-miR-148b |
| 60 | 0.6 | 0.0077233 | hsa-miR-24 |
| 61 | 0.6 | 0.0077233 | hsa-miR-576-3p |
| 62 | 0.598 | 0.0079409 | hsa-miR-199a-3p |
| 63 | 0.598 | 0.0079409 | hsa-miR-324-3p |
| 64 | 0.598 | 0.0079409 | hsa-miR-502-3p |
| 65 | 0.596 | 0.0081635 | hsa-miR-299-3p |
| 66 | 0.596 | 0.0081635 | hsa-miR-29a |
| 67 | 0.596 | 0.0081635 | hsa-miR-345 |
| 68 | 0.596 | 0.0081635 | hsa-miR-374b |
| 69 | 0.595 | 0.0083912 | hsa-miR-18a |
| 70 | 0.595 | 0.0083912 | hsa-miR-20b |
| 71 | 0.595 | 0.0083912 | hsa-miR-335 |
| 72 | 0.595 | 0.0083912 | hsa-miR-518e |
| 73 | 0.595 | 0.0083912 | hsa-miR-744 |
| 74 | 0.593 | 0.008624 | hsa-miR-452 |
| 75 | 0.591 | 0.0088621 | hsa-miR-886-3p |
| 76 | 0.591 | 0.0088621 | hsa-miR-92a |
| 77 | 0.59 | 0.0091055 | hsa-miR-152 |
| 78 | 0.59 | 0.0091055 | hsa-miR-339-3p |
| 79 | 0.59 | 0.0091055 | hsa-miR-501-3p |
| 80 | 0.59 | 0.0091055 | hsa-miR-523 |
| 81 | 0.59 | 0.0091055 | hsa-miR-598 |
| 82 | 0.588 | 0.0093543 | hsa-miR-185 |
| 83 | 0.588 | 0.0093543 | hsa-miR-195 |
| 84 | 0.588 | 0.0093543 | hsa-miR-204 |
| 85 | 0.586 | 0.0096086 | hsa-miR-224 |
| 86 | 0.584 | 0.0098685 | hsa-miR-146a |
| 87 | 0.584 | 0.0098685 | hsa-miR-15b |
| 88 | 0.584 | 0.0098685 | hsa-miR-326 |
