# Supplementary figures and images for: miR-203 and miR-221 regulate SOCS1 and SOCS3 in essential thrombocythemia
Source: Blood Cancer J. 2016 Mar 18;6(3):e406–. doi: 10.1038/bcj.2016.10 (PMC4817095; doi:10.1038/bcj.2016.10)

## Slide 1
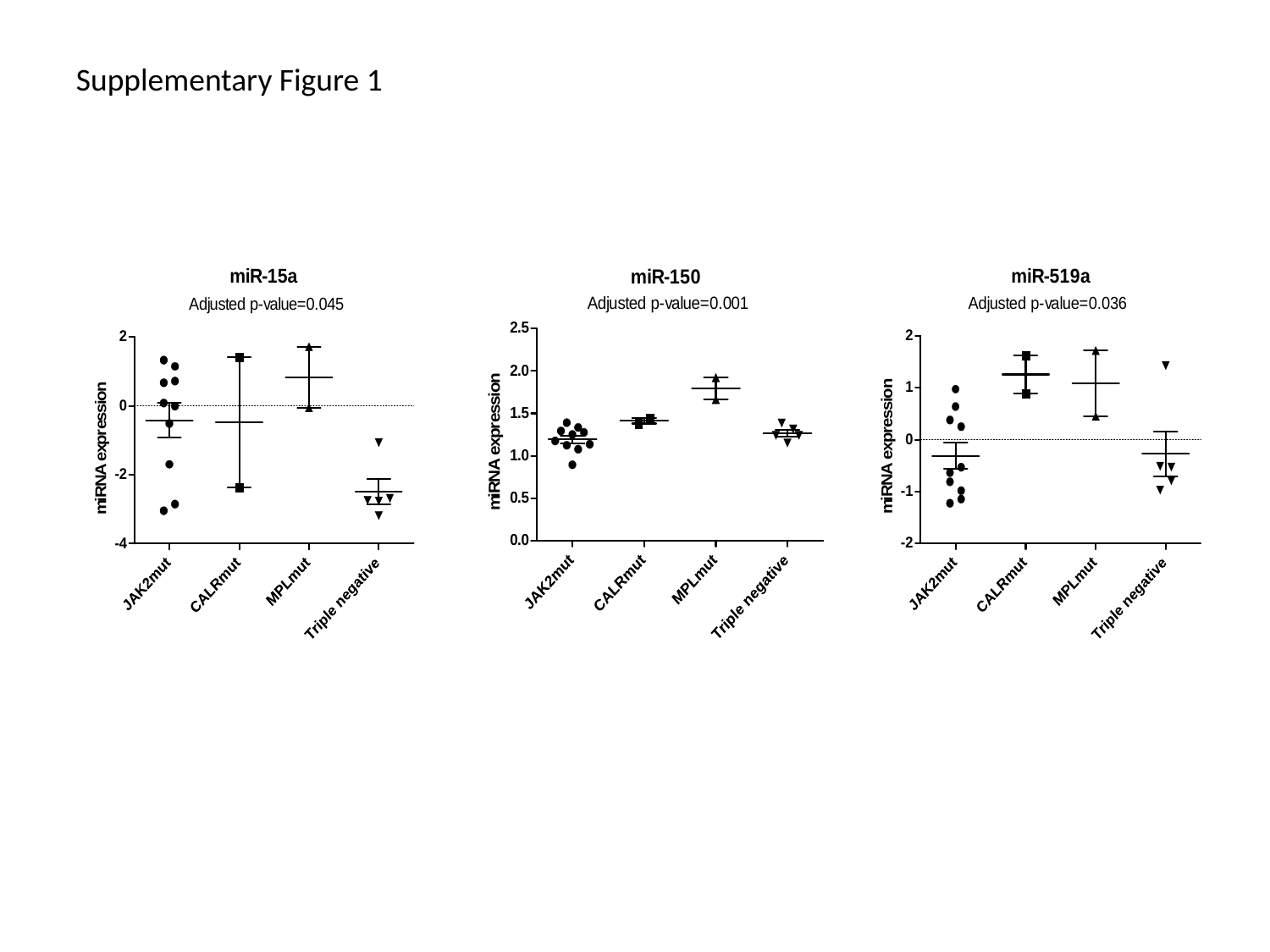

Supplementary Figure 1

Supplement: Supplementary Figure 1 [file bcj201610x4.ppt]
