# Supplementary material for: miR-203 and miR-221 regulate SOCS1 and SOCS3 in essential thrombocythemia
Source: Blood Cancer J. 2016 Mar 18;6(3):e406–. doi: 10.1038/bcj.2016.10 (PMC4817095; doi:10.1038/bcj.2016.10)
Supplement: Supplementary Figure Legend [file bcj201610x5.docx]

**Supplementary Figure 1. MiRNAs differentially expressed according mutational status of *JAK2*, *CALR* and *MPL*.** Three miRNAs were identified using ANOVA: miR-15a, miR-150 and miR-519a.
